# Smoking among Brazilian adolescents during the COVID-19 pandemic: a cross-sectional study

**DOI:** 10.1590/1516-3180.2022.0424.R1.30032023

**Published:** 2023-05-26

**Authors:** Deborah Carvalho Malta, Crizian Saar Gomes, Nádia Machado de Vasconcelos, Francielle Thalita Almeida Alves, Arthur Pate de Souza Ferreira, Marilisa Berti de Azevedo Barros, Margareth Guimarães Lima, Celia Landmann Szwarcwald

**Affiliations:** I PhD. Physician and Associated Professor, Department of Maternal and Child Nursing and Public Health, Faculty of Nursing, Universidade Federal de Minas Gerais (UFMG), Belo Horizonte (MG), Brazil.; II PhD. Nutritionist and Post-doctoral Student, Postgraduate Program in Public Health, Faculty of Medicine, Universidade Federal de Minas Gerais (UFMG), Belo Horizonte (MG), Brazil.; III MSc. Physician and Doctoral Student, Postgraduate Program in Public Health, Faculty of Medicine, Universidade Federal de Minas Gerais (UFMG), Belo Horizonte (MG), Brazil.; IV Undergraduate Student, Faculty of Nursing, Universidade Federal de Minas Gerais (UFMG), Belo Horizonte (MG), Brazil.; V PhD. Researcher, Department of Epidemiology and Quantitative Methods in Health, Fundação Oswaldo Cruz, Rio de Janeiro (RJ), Brazil.; VI PhD. Physician and Full Professor, Department of Public Health, Faculty of Medical Sciences, Universidade Estadual de Campinas (UNICAMP), Campinas (SP), Brazil.; VII PhD. Researcher, Department of Public Health, Faculty of Medical Sciences, Universidade Estadual de Campinas (UNICAMP), Campinas (SP), Brazil.; VIII PhD. Senior Researcher, Laboratório de Informação e Saúde (LIS), Instituto de Comunicação e Informação Científica e Tecnológica em Saúde (ICICT), Fundação Oswaldo Cruz (Fiocruz), Rio de Janeiro (RJ), Brazil.

**Keywords:** Tobacco use, Adolescent, COVID-19, Cross-sectional studies, Brazil, Health inequality monitoring, Health surveys, Tobacco dependence

## Abstract

**BACKGROUND:**

The social distancing measures during the coronavirus disease 2019 (COVID-19) pandemic resulted in mental suffering among adolescents, leading to risky consumption of psychoactive substances such as tobacco.

**OBJECTIVE:**

To analyze the factors associated with tobacco use among adolescents during the COVID-19 social distancing period in Brazil.

**DESIGN AND SETTING:**

Cross-sectional study used data from ConVid Adolescentes survey in Brazil.

**METHODS:**

Tobacco use was assessed before and during social distancing. The explanatory variables investigated were sex, age, race/skin color, type of school, maternal education, region of residence, adherence to social restriction measures, number of close friends, sleep quality during the pandemic, mood, passive smoking, use of alcoholic beverages during the pandemic, sedentary behavior, and physical activity. A logistic regression model was used for the data analysis.

**RESULTS:**

Tobacco use by adolescents did not change during the pandemic (from 2.58% to 2.41%). There was a higher chance of tobacco use among adolescents aged between 16 and 17 years, self-reported black ones, residing in the South and Southeast regions, reported feeling sad and loneliness, had sleeping problems that worsened, were using alcoholic beverages during the pandemic, and were passive smokers at home. Adolescents whose mothers had completed high school or higher, had strict social restrictions, and increased their physical activity during the pandemic had a lower chance of tobacco use.

**CONCLUSION:**

Tobacco uses during the COVID-19 pandemic was higher in vulnerable groups, such as black adolescents and those with mental suffering.

## INTRODUCTION

The coronavirus disease 2019 (COVID-19) was declared a pandemic in March 2020 by the World Health Organization. In several Brazilian states and cities, social distancing measures were decreed to reduce the spread of the disease, such as class suspension, non-essential commerce closure, and travel restriction.^1,2,3^ Studies have described the repercussions of reduced social interaction, pointing to an increase in stress, loneliness, and sadness,^4^ as well as a worsening in lifestyles.^5^ Thus, studies have identified that mental suffering and feelings such as anxiety, loneliness, and sadness can lead to consumption of risky substances such as alcohol and tobacco.^5,6,7,8,9^

Tobacco use among adolescents is a global health problem. A Global Youth Tobacco Survey review conducted in 131 countries/territories between 1999 and 2005 found that 8.9% of adolescents aged 13–15 years had smoked on one or more days during the last 30 days.^10^ Tobacco use can result in health damage,^11^ including an increase in adult mortality among those who begin smoking in childhood and adolescence, compared to never smokers.^10^

Studies have shown that the prevalence of tobacco use is decreasing in Brazil and worldwide,^12,13^ however, among adolescents, this issue remains a Public Health concern, given the high prevalence of consumption of other products such as hookah and electronic cigarettes among Brazilian students.^14,15^

The “ConVid Comportamentos” Study identified that the prevalence of adult smokers was 12% (confidence interval, CI 95%: 11.1–12.9) during the pandemic, of which 34% reported an increase in cigarette consumption. This increase in Brazilian adults was greater among women and individuals with incomplete secondary education. The increase in cigarette consumption among Brazilian adults was associated with worse sleep quality, feeling sad or depressed, anxiety, feeling isolated from family members, having no income, and worse health status.^5^ A study in Spain showed that smoking during the pandemic was used by adolescents to relieve unpleasant emotions.^16^

Among Brazilian adolescents, a study analyzing data from the ConVid Adolescentes Survey showed a decrease in tobacco consumption.^17^ However, the factors associated with tobacco consumption during the pandemic have not yet been analyzed.

## OBJECTIVE

This study aimed to analyze the factors associated with tobacco use among adolescents during the social distancing period in Brazil during the COVID-19 pandemic.

## METHODS

A cross-sectional study that analyzed the database of “ConVid Adolescentes – Research of Behaviors” survey was conducted with Brazilian adolescents aged 12–17 years. ConVid Adolescentes is a virtual health survey aimed at evaluating changes in adolescents’ lives due to the COVID-19 pandemic.

Data collection from ConVid Adolescentes was conducted via the Internet, using a self-completion questionnaire via a cell phone or computer, and took place between June 27 and October 12, 2020. The questionnaire was constructed using the Research Electronic Data Capture application and included questions about sociodemographic characteristics and changes in lifestyle, routine activities, mood, and family relationships during the social distancing period (https://convid.fiocruz.br/index.php?pag=questionario_adolescente). The information was stored on the server at the Institute of Communication and Scientific and Technological Information in Health of the Oswaldo Cruz Foundation (Instituto de Comunicação e Informação Científica e Tecnológica em Saúde, Fundação Oswaldo Cruz [ICICT/FIOCRUZ]).

Participants were invited through a chain sampling procedure, called a virtual “snowball”.^18^ First, the link of the research was sent to researchers from different states of Brazil, with previous experience in studies with adolescents. Theses researchers sent the link to other adults in their social network with adolescent children. These adults were then asked to invite at least three more parents or guardians of adolescent children. Thus, invitations were sent to adults and, upon receiving the invitation to participate in the research, they were asked, “Do you have children or are you responsible for young people aged between 12 and 17 years old?”. Only those who answered affirmatively received the Free and Informed Consent Term with explanations about the survey, a link to contacts and clarification about the research, and a request for consent to participation of the minor under their responsibility. After obtaining the consent of the responsible adult, the adolescent received the Free and Informed Assent Term and completed the questionnaire. In addition, the research coordinator sent letters to the State Department and schools inviting them to send the link to parents and adolescents. The final sample consisted of 9,470 adolescents aged 12–17 years.

Because sampling by networking is not probabilistic, to obtain a representative sample of the population according to geographic and sociodemographic characteristics, weights were calculated using post-stratification procedures.^19^ The sample was calibrated using data from the National School Health Survey,^20^ and aimed to obtain the same distribution by region of residence, sex, age group (12–15 years; 16–17 years), and type of school (public or private).

### Variables

In the present study, tobacco consumption before and during the pandemic was analyzed by asking the following questions: a) Before the pandemic, did you smoke cigarettes? (Yes or No); b) During the pandemic? 1- I did not smoke cigarettes; 2- I am smoking less than I used to; 3- I continued to smoke at the same frequency; 4- I am smoking more than I used to; 5- I stopped smoking but I started smoking again. Adolescents who answered “yes” to the question before the pandemic and options 2, 3, 4 or 5 during the pandemic were considered smokers.

The following explanatory variables were investigated: sex (female and male), age group (12–15 years; 16–17 years), race/skin color (white; black; brown; others), type of school (public and private), maternal education (elementary school or less; complete high school; complete higher education), region of residence (North, Northeast, Southeast, South and Midwest), adherence to social restriction measures (not very strict and very strict), number of close friends (none, 1 friend, 2 friends, 3 or more friends), sleeping quality during the pandemic (did not affect, began having sleeping problems, continued to have sleeping problems, sleeping problems got worse, reduced sleeping problems), mood [feeling sad or depressed (never/rarely, sometimes, always); feeling irritated (never/rarely, sometimes, always); feeling isolated (never/rarely, sometimes, always)], passive smoking (yes and no), consumption of alcoholic beverages during the pandemic (yes and no), sedentary behavior (maintained, increased, reduced), physical activity (maintained, increased, reduced).

### Statistical analyzes

Initially, the prevalence and 95% confidence interval (CI) of cigarette consumption before and during the pandemic were calculated for the total sample according to the explanatory variables.

To verify the possible factors associated with smoking during the pandemic, crude and adjusted odds ratios (ORc and ORa, respectively) by sex, age group, and type of school logistic regression models were used, with a significance level of 5%. All analyses were performed using the Software for Statistics and Data Science (StataCorp LP, CollegeStation, Texas, United States), version 14.0, and post-stratification weights were considered.

### Ethical issues

This study was approved by the National Research Ethics Committee (opinion no.: 4,100,515, June 20, 2020). The parents or guardians of the adolescents completed a Free and Informed Consent Form, followed by their own consent. None of the adolescents were identified.

## RESULTS

A total of 9,470 adolescents were evaluated, of which 50.25% (95% CI: 48.58–51.91) were female and 67.68% (95% CI: 66.28–69.05) were aged between 12–15 years. Most adolescents self-reported being of the race/color of skin brown (46.6%; 95% CI: 44.91–48.26), followed by white (40.10%; 95% CI: 38.53–41.60) and studying in public schools (85.90%; 95% CI: 85.12–86.70). The distribution of maternal education was similar, with approximately one-third of the participants in each group (Table 1).

**Table 1.** Sample characteristics. ConVid Adolescentes, 2020. (n = 9,470)

Variables	Percentage (%)	95% CI
**Sex**		
Male	49.75	48.09–51.42
Female	50.25	48.58–51.91
**Age group**		
12 to 15 years old	67.68	66.28–69.05
16 and 17 years old	32.32	30.95–33.72
**Race/skin color**		
White	40.09	38.53–41.66
Black	9.70	8.76–10.73
Brown	46.58	44.91–48.26
Others	3.63	3.01–4.38
**Type of school**		
Private	14.07	13.3–14.88
Public	85.93	85.12–86.7
**Maternal education**		
Elementary school or less	32.57	30.94–34.24
Complete high school	33.80	32.13–35.51
Complete higher education	33.63	32.06–35.23

CI = confidence interval. Complete higher education: undergraduated or higher.

**Figure 1.**
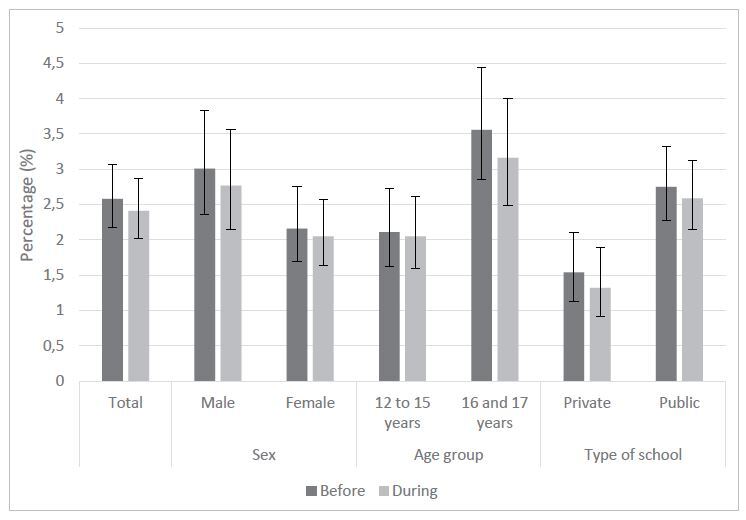
Prevalence and 95% confidence interval of smoking before and during the coronavirus disease 2019 (COVID-19) pandemic. ConVid Adolescentes, 2020.

Cigarette consumption was reported by 2.58% (95% CI: 2.17–3.07) of adolescents before the pandemic and remained unchanged during the pandemic (2.41%; 95% CI: 2.02; 2.87). There was no statistically significant difference in the prevalence of cigarette consumption before and during the pandemic between sexes, age groups, and type of school (Figure 1).

When analyzing the factors associated with tobacco consumption during the pandemic, it was observed that older adolescents (ORa: 1.55; 95% CI: 1.09–2.20), who self-reported as black (ORa: 1.82; 95% CI: 1.05–3.15), who lived in the Southeast (ORa: 4.33; 95% CI: 2.55–7.36) and South (ORa: 2.33; 95% CI: 1.37–3.96), who used alcoholic beverages during the pandemic (ORa: 19.9; 95% CI: 13.10–30.20), who were passive smokers at home (ORa: 4.68; 95% CI: 3.26–6.71), who reported that sleeping problems got worse during the pandemic (ORa: 2.37; 95% CI: 1.49–3.76), who always felt sad (ORa: 1.91; 95% CI: 1.15–3.17) and who sometimes (ORa: 1.71; 95% CI: 1.05–2.80) or always (ORa: 1.65; 95% CI: 1.04–2.62) felt lonely, were more likely to consume tobacco during the pandemic. On the other hand, adolescents who study in private schools (ORa: 0.52; 95% CI: 0.34–0.78),whose mothers had completed high school (ORa: 0.33; 95% CI: 0.21–0.53) or higher (ORa: 0.45; 95% CI: 0.27–0.75), who had very strict social restrictions (ORa: 0.30;95% CI: 0.20–0.43) and that physical activity increased during de pandemic (ORa: 0.40; 95% CI: 0.16–0.98), were less likely to consume tobacco (Table 2).

**Table 2.** Prevalence and crude and adjusted odds ratio (OR) (95% confidence interval [CI]) of smoking by adolescents during the pandemic, according to potential associated factors. ConVid Adolescentes, 2020

Variables	During % (95% CI)	Crude OR (95% CI)	Adjusted*OR (95% CI)
**Total**	2.41 (2.02; 2.87)		
**Sex**			
Male	2.77 (2.15; 3.57)	–	–
Female	2.05 (1.64; 2.57)	0.74 (0.52; 1.04)	0.74 (0.52; 1.04)
**Age group**			
12 to 15 years old	2.05 (1.60; 2.62)	–	–
16 and 17 years old	3.16 (2.49; 4.00)	1.56 (1.10; 2.22)	1.55 (1.09; 2.20)
**Race/Skin color**			
White	2.09 (1.57; 2.77)	–	–
Black	4.09 (2.60; 6.38)	2.00 (1.15; 3.46)	1.82 (1.05; 3.15)
Brown	2.34 (1.79; 3.06)	1.12 (0.75; 1.67)	1.05 (0.70; 1.58)
Others	2.48 (1.13; 5.36)	1.19 (0.51; 2.79)	1.12 (0.48; 2.64)
**Type of school**			
Public	2.59 (2.15; 3.12)	–	–
Private	1.32 (0.91; 1.89)	0.50 (0.33; 0.76)	0.52 (0.34; 0.78)
**Maternal Education**			
Elementary school or less	4.22 (3.27; 5.42)	–	–
Complete high school	1.41 (0.96; 2.06)	0.32 (0.20; 0.52)	0.33 (0.21; 0.53)
Complete higher education	1.78 (1.25; 2.54)	0.41 (0.26; 0.64)	0.45 (0.27; 0.75)
**Region of residence**			
North	1.02 (0.64; 1.63)	–	–
Northeast	0.46 (0.15; 1.38)	0.45 (0.14; 1.50)	0.45 (0.13; 1.49)
Southeast	4.15 (3.34; 5.15)	4.20 (2.48;7.11)	4.33 (2.55; 7.36)
South	2.35 (1.86; 2.96)	2.34 (1.37; 3.98)	2.33 (1.37; 3.96)
Midwest	2.07 (1.12; 3.79)	2.05 (0.94; 4.49)	2.12 (0.97; 4.62)
**Social restriction**			
Not very strict	4.84 (3.82; 6.10)	–	–
Very strict	1.44 (0.10; 1.87)	0.29 (0.20; 0.41)	0.30 (0.20; 0.43)
**Close friends**			
None	1.83 (1.05; 3.18)	–	–
1 friend	2.63 (1.65; 4.16)	1.45 (0.69; 3.02)	1.49 (0.71; 3.09)
2 friends	2.59 (1.81; 3.70)	1.42 (0.73; 2.79)	1.49 (0.76; 2.92)
3 or more friends	2.36 (1.85; 3.00)	1.30 (0.70; 2.40)	1.38 (0.74; 2.57)
**Sleeping**			
Did not affect	1.96 (1.40; 2.57)	–	–
Started to have sleeping problems	2.66 (1.84; 3.83)	1.37 (0.86; 2.19)	1.43 (0.89; 2.31)
Continued to have sleeping problems	1.30 (0.73; 2.28)	0.66 (0.35; 1.24)	0.67 (0.36; 1.27)
Sleeping problems got worse	4.33 (3.10; 6.03)	2.27 (1.45; 3.55)	2.37 (1.49; 3.76)
Reduced sleeping problems	4.54 (2.01; 9.95)	2.38 (0.98; 5.79)	2.40 (0.99; 5.86)
**Feeling sad/depressed**			
Never/rarely	1.79 (1.26; 2.54)	–	–
Sometimes	2.52 (1.85; 3.42)	1.42 (0.88; 2.29)	1.53 (0.92; 2.56)
Always	2.94 (2.26; 3.83)	1.67 (1.06; 2.61)	1.91 (1.15; 3.17)
**Feeling irritated**			
Never/rarely	2.26 (0.49; 3.40)	–	–
Sometimes	2.46 (1.78; 3.39)	1.09 (0.64; 1.87)	1.15 (0.66; 1.98)
Always	2.45 (1.93; 3.11)	1.09 (0.67; 1.77)	1.18 (0.69; 2.01)
**Feeling isolated**			
Never/rarely	1.82 (1.32; 2.51)	–	–
Sometimes	2.85 (2.08; 3.90)	1.58 (1.00; 2.51)	1.71 (1.05; 2.80)
Always	2.74 (2.08; 3.61)	1.52 (0.98; 2.34)	1.65 (1.04; 2.62)
**Passive smoking**			
No	1.42 (1.12; 1.79)	–	
Yes	6.40 (4.96; 8.22)	4.76 (3.32; 6.82)	4.68 (3.26; 6.71)
**Consumption of alcoholic beverages during the pandemic**			
No	0.83 (0.60; 1.14)	–	–
Yes	13.30 (10.90; 16.12)	18.33 (12.4; 27.14)	19.90 (13.10; 30.20)
**Sedentary behavior**			
Maintained	2.23 (1.79; 2.78)	–	–
Increased	2.91 (2.15; 3.93)	1.31 (0.90; 1.93)	1.31 (0.89; 1.92)
Reduced	2.02 (0.75; 5.36)	0.91 (0.32; 2.55)	0.89 (0.32; 2.49)
**Physical practice**			
Maintained	2.36 (1.92; 2.91)	–	–
Reduced	2.82 (1.99; 3.99)	1.20 (0.79; 1.82)	1.16 (0.76; 1.76)
Increased	0.96 (0.4; 2.28)	0.40 (0.16; 0.99)	0.40 (0.16; 0.98)

* Adjusted for sex, age group, and school type.

## DISCUSSION

The “Convid Adolescentes” survey evaluated the changes in the lifestyle of Brazilian adolescents during social distancing of the COVID-19 pandemic. This study shows that tobacco consumption did not change during the pandemic. Higher consumption was associated with older adolescents, black race/color of the skin, residing in the South and Southeast regions, who reported feelings of sadness and loneliness, whose sleeping problems worsened, who consumed alcoholic beverages during the pandemic, and who reported passive smoking. Tobacco consumption was lower among adolescents whose mothers had higher educational levels, who studied in private schools, who adopted stricter social distancing measures during the pandemic, and who increased their physical activity during the pandemic.

This study demonstrated the stability of smoking habits among adolescents during the pandemic. Previous studies found the same pattern.^16,21^ On one hand, the stress and anxiety produced by the period of social isolation can be considered triggers for unhealthy behaviors and some adolescents could use smoking as a coping mechanism for these adverse feelings.^22^ On the other hand, the decrease in income and the closure of establishments aimed at social interaction, such as bars and restaurants, may have reduced adolescents’ access to cigarettes.^23,24^ Thus, more studies are needed to better understand the reasons for the stability of this prevalence.

Notably, the prevalence in the current study was lower than that in Spain^16^ and that in the data from the National Survey of School Health (Pesquisa Nacional de Saúde do Escolar [PeNSE]) 2019, which showed that tobacco consumption in the last 30 days was 6.8% (6.3–7.3) for students aged 13–17 years.^25^ The results showed a higher prevalence of tobacco consumption in older adolescents, which is in line with other studies.^16^ In PeNSE 2019, for example, it was found that adolescents aged 16–17 years consume more tobacco than those aged between 13–15 years.^25^

Adolescents who reported feelings of sadness, loneliness, and worsened sleep problems during the pandemic had higher tobacco consumption. Studies carried out during the pandemic have suggested that situations of social distancing can have negative consequences on psychological and mental health.^26–29^ Therefore, some adolescents may have been involved with substance use as a way to deal with psychological discomfort and negative feelings related to the COVID-19 situation.^27,28,30,31^ The increase in tobacco consumption in some groups may be a way to relieve negative emotions related to COVID-19, deal with boredom, and overcome the lack of social relationships.^27,28,30,32,33^

Lower tobacco consumption was observed among children of more educated mothers, which was also observed in a study from Spain for adolescents whose parents had a university education.^16^ The COVID-19 pandemic has led to social restrictions, and adolescents spent more time with their parents at home. Higher maternal education may be related to greater access to information, not only on the risks of tobacco but also on the possible respiratory aggravations that COVID-19 can generate in smokers.^16,30^ Thus, a family attitude of disapproval of tobacco consumption by adolescents at home may have contributed to this reduction.

Smoking was higher among Black adolescents, which may reflect their greater vulnerability. In Brazil, race/skin color is associated with lower income and may also be associated with parents with less education, which would lead to less access to information.^16^

Passive smoking had the strongest association among adolescents. This finding was also identified in a previous study with data from PeNSE 2015,^14^ among other studies.^34^ Passive smoking at home denotes the marked influence of family members and close people, and which are highlighted by the theory of social learning.^35^ Thus, adolescents frequently exposed to passive smoking naturalize the practice and end up adopting it.

The association between alcohol consumption and a greater chance of smoking during the pandemic may be explained by the fact that both habits are seen as a source of social acceptance,^36^ in addition to being risky behaviors based on the same vulnerabilities, such as feelings of sadness and isolation.^37^ Furthermore, a previous study showed that the consumption of alcoholic beverages increases the desire to smoke in people who already consume cigarettes.^38^

In line with this study, data from PeNSE 2019 show that the prevalence of tobacco use in the last 30 days was also higher in the South region of the country (8.0%; 95% CI: 7.1–8.7) and Southeast (7.6%; 95% CI: 6.4–8.7), compared to the North (4.7%; 95% CI: 4.3–5.2).^25^

Previous studies have suggested that the tobacco control measures implemented in the country were critical in the decline of smoking prevalence among adults^40^ and adolescents.^14^ Brazil has the lowest smoking prevalence among adolescents in America.^40^ Law n^o^ 12.546/2011,^41^ Presidential Decree no. 8.262/2014^42^ e an Interministerial Ordinance no. 2.647/2014^43^ forbidden advertising at sale’s point, determined the increase of prices and taxes, established closed environments completely smoke-free, and increasing images of health warnings, in addition to prohibiting the use of hookah.

However, in recent years, there have been setbacks in surveillance and regulatory policies, jeopardizing the health of children and adolescents. Data from PeNSE 2019 indicate a very high prevalence of hookah and electronic cigarette use, even though the latter is prohibited by the Brazilian Health Regulatory Agency (Agência Nacional de Vigilância Sanitária [ANVISA]).^25^

Among the limitations of the present study, it is worth mentioning that a non-random sample was selected via the Web, which may not have reached all social segments, although post-stratification weights were applied. The data obtained were based on reports by adolescents, which may have led to information bias. Furthermore, this is a cross-sectional study, which does not allow the establishment of a cause-effect relationship between the associations observed.

## CONCLUSIONS

The results suggest that the COVID-19 pandemic has affected the social lives of young people. Although there was no change in tobacco consumption, it is necessary to remain alert, especially in older black adolescents who are subjected to passive smoking at home, have mental suffering, and have less-educated parents.
